# A multi-centre real-world evaluation of AI-assisted organ at risk contouring on radiotherapy treatment planning workflows

**DOI:** 10.1093/bjr/tqag111

**Published:** 2026-05-15

**Authors:** Catriona Inverarity, Benjamin Caswell-Midwinter, Babak Jamshidi, Man Ting Kwong, Chloe Black, Akashdeep Singh Chauhan, Fowzia Ibrahim, Ami Mehta, Kim Fell, Mark Gooding, Chris Walker, Keith Langmack, Alison Griffiths, Salma Ayis, Juan I Baeza, Jon Hindmarsh, Angie A Kehagia, Anna Barnes

**Affiliations:** King’s Technology Evaluation Centre, London Institute for Healthcare Engineering, King’s College London, London SE1 7AR, United Kingdom; King’s Technology Evaluation Centre, London Institute for Healthcare Engineering, King’s College London, London SE1 7AR, United Kingdom; King’s Technology Evaluation Centre, London Institute for Healthcare Engineering, King’s College London, London SE1 7AR, United Kingdom; King’s Technology Evaluation Centre, London Institute for Healthcare Engineering, King’s College London, London SE1 7AR, United Kingdom; Medical Physics and Clinical Engineering, Medical Physics, Guy’s and St Thomas’ NHS Foundation Trust, London SE1 9RT, United Kingdom; King’s Technology Evaluation Centre, London Institute for Healthcare Engineering, King’s College London, London SE1 7AR, United Kingdom; Medical Physics and Clinical Engineering, Medical Physics, Guy’s and St Thomas’ NHS Foundation Trust, London SE1 9RT, United Kingdom; King’s Technology Evaluation Centre, London Institute for Healthcare Engineering, King’s College London, London SE1 7AR, United Kingdom; Centre for Rheumatic Diseases, Department of Inflammation Biology, School of Immunology and Microbial Sciences, Faculty of Life Sciences and Medicine, King’s College London, London SE5 9RJ, United Kingdom; Cancer National Programme of Care, Specialised Commissioning, NHS England, London, United Kingdom; Specialised Commissioning Team, NHS England, London SE1 8UG, United Kingdom; Inpictura Ltd, Abingdon OX14 3PX, United Kingdom; Division of Cancer Sciences, Faculty of Biology, Medicine and Health, The University of Manchester, Manchester M20 4BX, United Kingdom; Radiotherapy Physics, Newcastle Upon Tyne Hospitals NHS Foundation Trust, Newcastle Upon Tyne NE7 7DN, United Kingdom; Radiotherapy Physics, Nottingham University Hospitals NHS Trust, Nottingham NG5 1PB, United Kingdom; Research Economics and Consultancy in Healthcare Ltd., Solihull, United Kingdom; School of Life Course and Population Sciences, King’s College London, London SE1 1UL, United Kingdom; Department of Public Services Management & Organisation, King’s Business School, King’s College London, London WC2B 4BG, United Kingdom; Department of Public Services Management & Organisation, King’s Business School, King’s College London, London WC2B 4BG, United Kingdom; King’s Technology Evaluation Centre, London Institute for Healthcare Engineering, King’s College London, London SE1 7AR, United Kingdom; King’s Technology Evaluation Centre, London Institute for Healthcare Engineering, King’s College London, London SE1 7AR, United Kingdom

**Keywords:** AI-assisted contouring, auto-contouring, organs at risk, radiotherapy treatment planning, workflow optimization, acceptability, staff involvement, time savings, real-world evaluation, health technology assessment

## Abstract

**Objectives:**

This multicentre real-world evaluation, commissioned by NHS England, evaluated the impact of AI-assisted contouring of organs at risk on contour acceptability, workflows, and staffing.

**Methods:**

Data from 626 patients were collected from eight NHS radiotherapy departments. Time metrics from the date of planning CT scan to the start of first treatment were compared between manual and AI-enabled pathways. Acceptability scores for AI-generated contours were also collected.

**Results:**

AI-assisted contouring increased potential efficiency in treatment planning compared to manual methods, reducing time for contouring and redistributing workload across different staff types. 100% of manual contour reviews involved clinical oncologists compared to 38% reviewing AI-generated contours. However, workflow design meant that time saving was not observed across the whole treatment planning pathway. AI contours were generally well accepted, with 15.9% requiring no edits and 64.4% only minor edits. This varied by anatomy, with breast having the best acceptability and prostate and head and neck contours requiring more editing.

**Conclusions:**

AI contouring tools have the potential to enhance efficiency in radiotherapy treatment planning, creating operational flexibility. Pathway review and revision could unlock further benefits, for example, involving different staff types to address local bottlenecks. Workflow, capacity, and staffing review pre- and post-implementation could increase efficiency gains with AI-assisted contouring.

**Advances in knowledge:**

This study evaluated AI contouring tools in complex, real-world systems which differ between departments for generalizable conclusions. It describes the changes in time across the whole treatment planning pathway and the system-level impact of using AI tools to help inform holistic planning.

## Introduction

With cancer incidence rates rising in the UK,^1^^,^^2^ cancer services face growing demand for timely diagnosis and treatment. In 2021, there were 329,665 new cases diagnosed in England and 135,643 deaths from cancer.^3^^,^^4^ The top four primary tumour sites for radiotherapy treatments in England in 2021/22 were female breast, prostate, lung, and head and neck. The COVID-19 pandemic further exacerbated these challenges, leading to significant disruptions, in cancer referrals and treatment schedules.^5–7^

Alongside rising demand, chronic workforce shortages across radiography, medical physics and clinical oncology present a significant challenge for National Health Service (NHS) radiotherapy services.^8^^,^^9^ These shortages impact the capacity to meet rising demand for radiotherapy services, raising concerns over treatment delays, patient outcomes, and staff burnout.^10^^,^^11^ Workforce attrition can lead to a loss of experience, so the ability to standardize contouring can support consistency of care in resource-constrained systems. This can also support advanced level practice and career opportunity. Additionally, advances in radiotherapy treatments require time for adoption and may dictate more complex treatment planning, creating further pressures.

Considering these challenges, improving the efficiency of radiotherapy treatment planning while maintaining performance has become a priority. Contouring, the process enabling precise targeting of tumours while minimizing exposure to surrounding healthy tissues, is a time-consuming and labor-intensive step which is subject to intra- and inter-user variability.^12^^,^^13^ Specialist staff are required to manually delineate organs at risk (OARs) and the target volume (TV) on CT or MRI scans, which introduces variability due to differences in expertise, experience, and personal preferences among staff.^14–19^ Despite the involvement of other professionals in 65% of cases,^10^ clinical oncologists carry the primary responsibility for contouring. In response to these challenges, artificial intelligence (AI) has emerged as a promising solution in radiotherapy treatment planning.^20^

AI-enabled auto-contouring tools^21^ leverage deep learning (convolutional neural networks and generative adversarial networks) to automatically delineate OARs (and, in some cases, target volumes) and could reduce time required for treatment planning.^22^ This technology holds the potential to standardize practices across staff and departments, enable deployment of staff to address bottlenecks, and release time for other activities. Evidence collated by the National Institute for Health and Care Excellence (NICE)^23^ indicates that AI-generated contours achieve similar performance to manual ones, particularly for larger structures. It should be noted that most evidence exists for prostate and head and neck (H&N).

Existing literature suggests that AI contours are generally well accepted and often require only minor edits.^25^^,^^26^ However, performance varies across anatomies, with auto-contours of smaller, elongated structures often requiring more editing.^24^ Crowded anatomies and proximity to tumour sites can also affect performance.^27^ While AI-based auto-contouring could offer time savings,^28–31^ this depends on the quality and acceptability of the contour, including time for manual corrections, as well as the configuration of the AI-enabled workflow. Many studies only consider contouring times in isolation, without considering the entire workflow, and may therefore overestimate potential time savings across the whole treatment workflow.^23^^,^^32^

This multi-centre observational study was designed to evaluate the real-world effect of AI-assisted contouring of OARs on radiotherapy treatment planning compared to manual contouring (here, contours drawn entirely by radiotherapy or oncology staff without the use of AI, atlas-based, or other automated contouring tools), capturing variation in local workflows, staffing models, and pathway design. This approach aims to assess how AI-assisted contouring performs when deployed at scale in heterogeneous real-world clinical settings.

By measuring time in days rather than minutes, we aimed to capture impacts on the full treatment pathway, as the impacts of AI-assisted contouring could be offset by delays in other stages of the process. Therefore, the AI workflow is not solely defined by the AI’s performance, but how it integrates into and impacts the entire treatment planning workflow. Secondary objectives focused on evaluating the acceptability of AI-generated contours and the impact on staff involved.

## Methods

### Sites and population

Radiotherapy departments in eight NHS England Trusts participated in the study. AI software was implemented between 2020 and 2024. At the end of the evaluation period, two departments were using MVision (MVision AI Oy, Finland), two Limbus AI (Limbus AI, Canada), and two AI-Rad Companion Organs RT (Siemens Healthineers, Germany). Two departments submitted only manual contouring data. Several departments had previously used Mirada DLCExpert and were transitioning to alternatives following Mirada’s market exit. Two departments changed AI tools during the study. One provided data for both Siemens AI-Rad and Limbus AI during their transition, while the other exclusively supplied data for its new tool, Limbus AI. A third department reported the use of three different AI tools in consecutive years (though only one during the evaluation period). This department had a dedicated and audited AI-assisted pathway for stereotactic ablative body radiotherapy for lung cancer, supported by advanced practitioner radiographers. Only one other department reported conducting an audit and review of pathways after AI adoption.

No departments adopted AI pathways during the evaluation, so the best case of before-and-after comparison with departments was not possible. Reverting AI-using departments to human contouring was not feasible. Instead, manual contouring data were collected from two non-AI departments, and retrospective manual contouring data were gathered from departments using AI. While all NHS departments are subject to the same time-to-treatment standard,^33^ the application of efficiency gains may differ according to local service needs, including maintaining current performance with reduced workload, expanding capacity, or improving timely access to treatment.

### Data overview and sample

**Figure 1:**
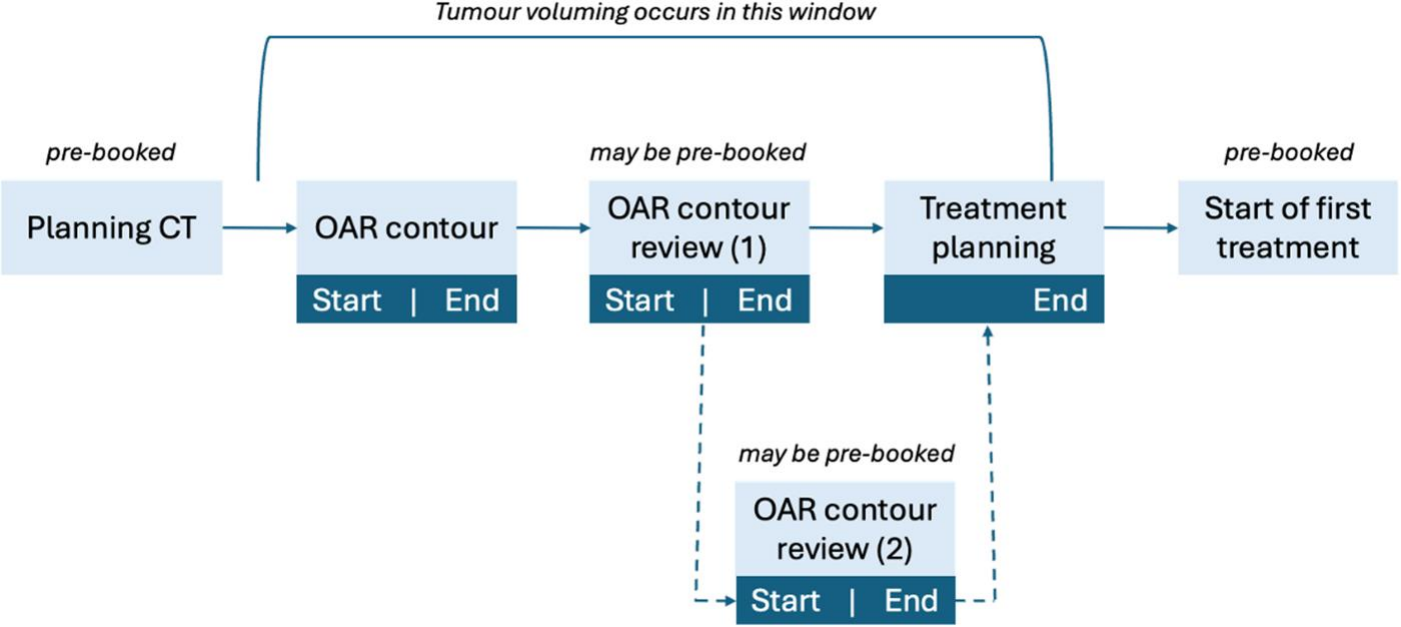
Outline of the treatment planning pathway summarising those described by departments. Pre-booked steps are ‘fixed’ at department level. All departments review OAR contours (“Reviewer 1”). Some departments have a second review step which may involve one or more members of staff (“Reviewer 2”).

A total of 626 contouring datasets (each representing an individual treatment plan and associated contours) were collected using validated spreadsheets. Each dataset captured the treatment planning process for a single patient, including workflow dates, the contouring agent (manual or AI), and the reviewer(s) (Figure 1). The following dates (DDMMYY) were recorded throughout the radiotherapy treatment process: referral date, planning CT date, OAR contour start, and end dates (the date on which contours of OARs were started and marked complete for each case), OAR contour review start and end dates (the date on which review of OAR contours were started and marked complete; with end date representing the point at which the last reviewer marked ‘complete’), radiotherapy treatment planning start and end dates (the date on which remaining treatment planning activities start and complete), and first treatment start date. These dates were all taken from timestamps generated in the oncology information systems at participating departments, supporting consistency of recording and reducing burden of data collection on departments.

From these dates, two categories of time metrics were derived. Sequential time intervals were defined as the elapsed time between consecutive workflow steps (e.g. planning CT to OAR contour completion, OAR contour completion to treatment planning completion). Cumulative workflow times were defined as the total elapsed time from planning CT to first treatment. The study focused on system-level workflow timing derived from routinely recorded clinical timestamps to reflect real-world pathway performance across multiple centres with heterogeneous workflows. Four primary tumour sites were represented (lung, head and neck, breast, and prostate), with breast and prostate additionally reported as separate “with nodes” groups where nodal volumes were included: lung (138 datasets, 22%), head and neck (114 datasets, 18%), breast (113 datasets, 18%), prostate (106 datasets, 17%), breast and nodes (68 datasets, 11%), and prostate and nodes (87 datasets, 14%). The datasets recorded whether contours were generated by AI or manually, as well as the professional role of the reviewer(s). Contours were reviewed using a 4-point scale based on Van Dijk et al.^34^ Departments with multiple reviewers provided the dates and reviewers’ profession for subsequent reviews.

**Figure 2:**
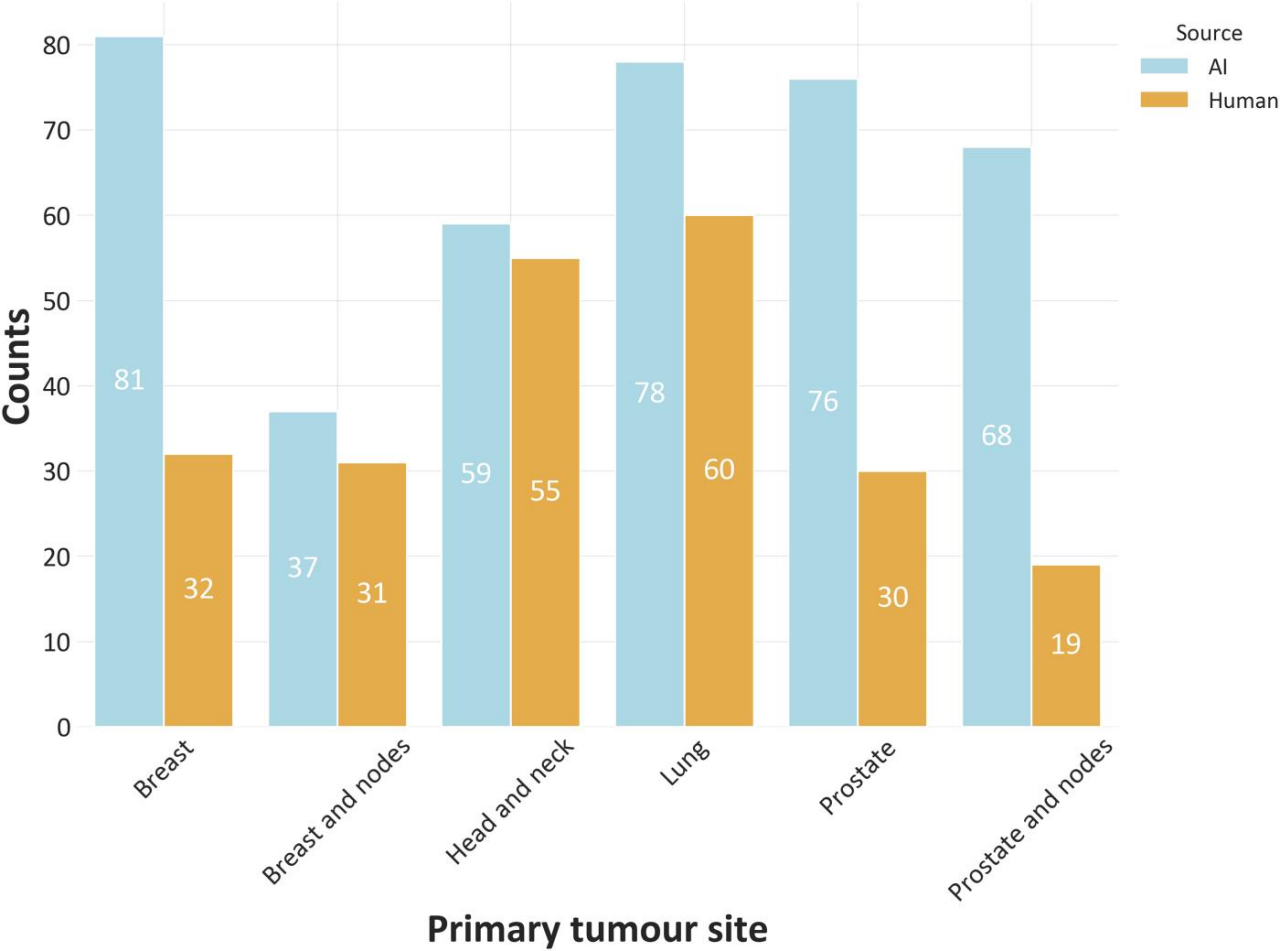
Bar plot of the number of tumour sites contoured according to the contouring agent.

Of the total, 399 datasets (63.7%) were AI-contoured at 6 departments and 227 were manually contoured at 6 departments. Figure 2 shows the distribution of tumour sites by contouring method. The datasets span 2020 to 2024, with the majority (429, 68.6%) from patients referred in 2024. The earliest referral date was September 2020 and the most recent was May 2024.

### Statistical analysis

AI-assisted and manual contouring time intervals were compared sequentially, analysing the time between consecutive steps (the time between each timestamp recorded from the workflow; Figure 1), and cumulatively (cumulative time from point of referral to treatment planning), measuring the time from the planning CT to later in the workflow. For non-parametric data, the Mann-Whitney U test was used to compare the two groups, with a significance level set at 0.05. Multivariate generalized linear models (GLMs) were used to evaluate the impact of department characteristics (i.e., number of staff, number of linear accelerators, tumour site) on two key time intervals: contouring start to contour review and acceptance (a sequential variable on contouring time) and planning CT to first treatment start (a cumulative variable across the pathway). A Poisson GLM was used for contouring time while a negative binomial GLM was applied for the time from planning CT to treatment start. Univariate analysis identified significant variables for inclusion in the multivariate GLM. GLMs examined the effects of contouring method (AI vs manual), tumour site, node inclusion, department, impact of COVID-19, adherence to new cancer waiting time standards,^33^ length of time AI contouring has been in use, staff-to-linear accelerator ratios, and the involvement of different staff types in treatment planning. A COVID-19 period variable was included to account for potential system-level disruption during the pandemic (defined as referrals between March 2020 and December 2023). Variables with *P*-values < .20 were included in the multivariate GLM.

For the statistical analysis of staff distribution and contour acceptability, descriptive statistics summarized the roles of staff involved in the workflows. The proportions of staff types performing first reviews of AI-generated contours were calculated and compared to manual workflows, where clinical oncologists exclusively reviewed contours.

The acceptability of AI-generated contours was also analysed. Chi-square tests were conducted to examine the association between tumour site and contour acceptability, with a significance level set at *P* < .05. Second reviews, where routinely performed in a local workflow, were also summarized to assess alignment with first reviewers. All analyses were performed using Python (version 3.9).

## Results

### Workflow duration

AI-assisted contouring significantly reduced the duration of the contouring activity (contour start to contour end), with AI-assisted cases completed on the same day (median = 0 days, interquartile range (IQR) = 0 days) compared with manual cases (median = 3 days; IQR = 2 days; U = 7,179, *P* < .001). The time from treatment planning start to plan sign-off was shorter in AI-assisted workflows (median = 1 vs 2 days; IQR = 3 days in both groups; U = 13,842, *P* < .001).

However, while AI expedited the contouring process itself, workflow structure meant that time savings were concentrated around contouring activities and not translated across the whole pathway. The interval between OAR contour review start and end was longer in the AI pathway (median = 3 days, IQR = 7 days) compared to the manual pathway (median = 1 day, IQR = 5 days; U = 60,474, *P* < .001). Additionally, the time from plan completion to treatment start (AI: median = 6 days, IQR = 5 days; manual: median = 5 days; IQR = 6 days; U = 55,085, *P* < .001) was longer in the AI-assisted workflow. The GLM indicated that contouring times were significantly longer during the COVID-19 period (β = 2.60E + 7, *P* < .001).

**Figure 3:**
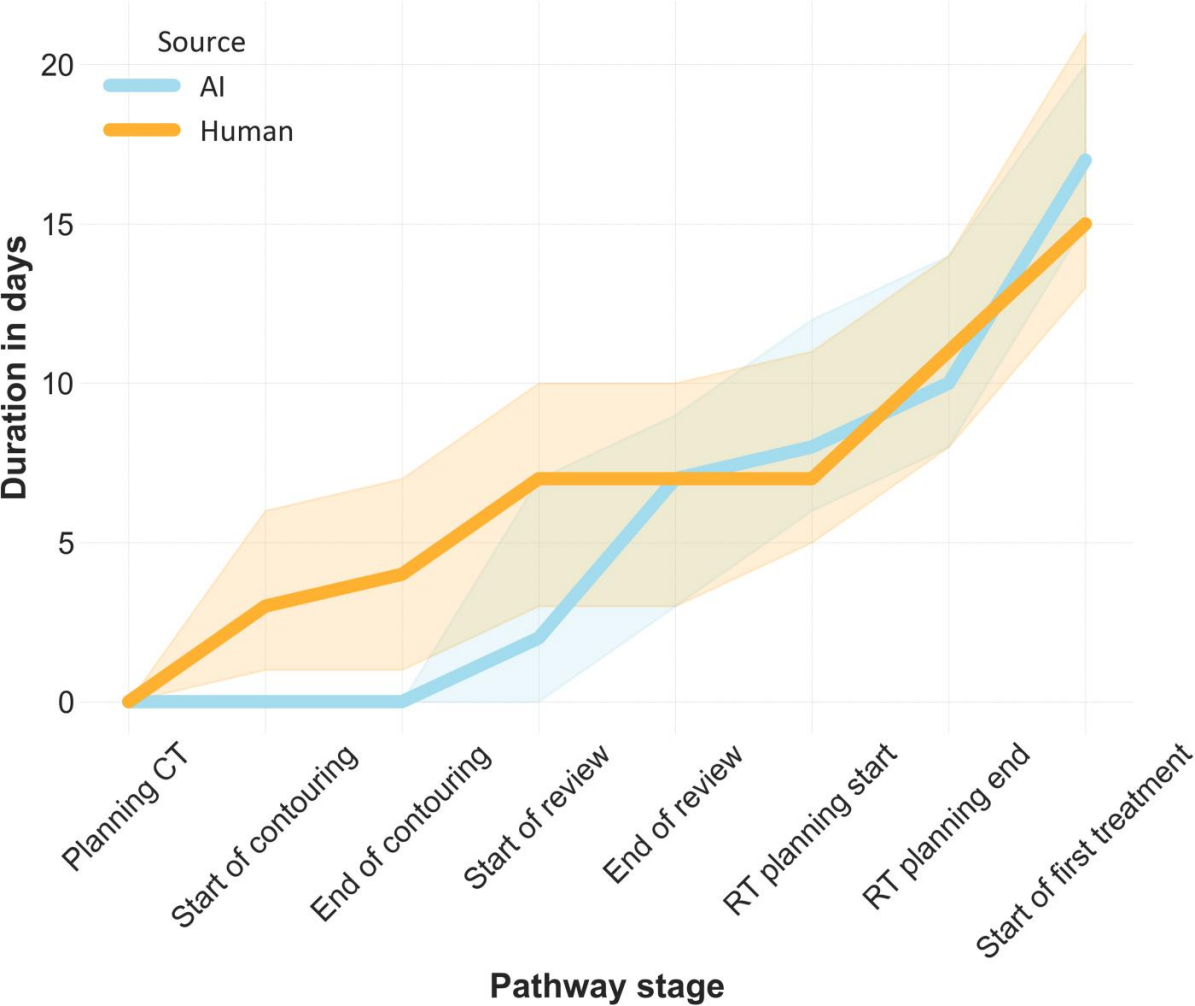
Line plot showing the median cumulative time required in the pathway from planning CT to the following steps with AI-generated and manual OAR contouring. Shading denotes the interquartile range (IQR).

Cumulative time interval analysis reinforced these findings (Figure 3), showing that while AI-assisted pathways reduced OAR contouring time, the overall pathway duration was similar to manual contouring. The AI-assisted pathways generally required less time from planning CT to contour review completion, in line with the sequential time interval results. The median time to start OAR contouring was 0 days for AI-assisted cases compared to 3 days for manual contouring (IQR = 5 days). Similarly, the median time to complete OAR contouring was 0 days for AI-assisted cases and 4 days for manual cases (IQR = 6 days).

By the end of the contour review, both AI and manual contouring pathways converged to a median of 7 days (AI-assisted IQR = 6 days, manual IQR = 7 days). Despite the initial time savings observed with AI, the overall median duration from planning CT to the start of treatment was similar between AI-assisted (17 days, IQR = 5 days) and manually contoured cases (15 days, IQR = 8 days), with no significant difference across tumour sites (β = 1.076, *P* = .407). While prostate treatments showed a trend towards shorter pathway times in univariate analysis (β = 1.228, *P* = .021), this was not significant in the GLM (*P* = .091).

### Distribution of staff roles involved in workflow

**Figure 4:**
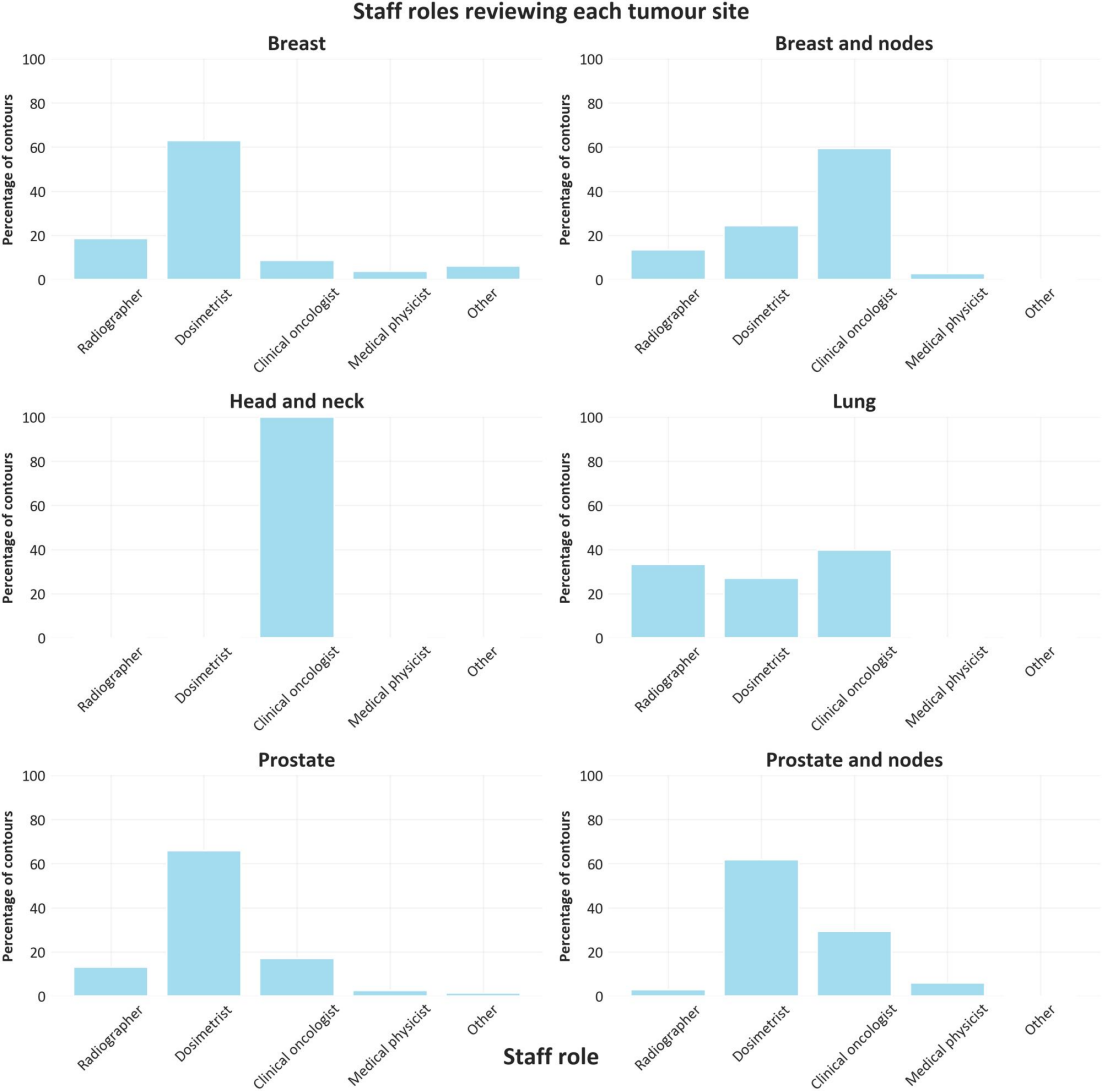
Staff breakdown by profession conducting the first reviews of the AI-generated contours.

Manual contouring was predominantly performed by clinical oncologists (86%), with therapeutic radiographers (12%) and dosimetrists (2%) contributing to fewer cases (and only at 3 of 6 departments who provided manual data). In the AI pathway, OAR contouring was done by AI. Reviewer 1 provided acceptability scores for 396 AI-generated contours (3 missing). Of these, 43% were first reviewed by dosimetrists, 38% by clinical oncologists, 14% by therapeutic radiographers, 3% by medical physicists, and 2% by advanced clinical practitioners. In contrast, all manual contours were reviewed exclusively by clinical oncologists. Figure 4 displays the distribution of reviewer roles by tumour site for AI-generated contours.

### Acceptability of AI-generated contours

**Figure 5:**
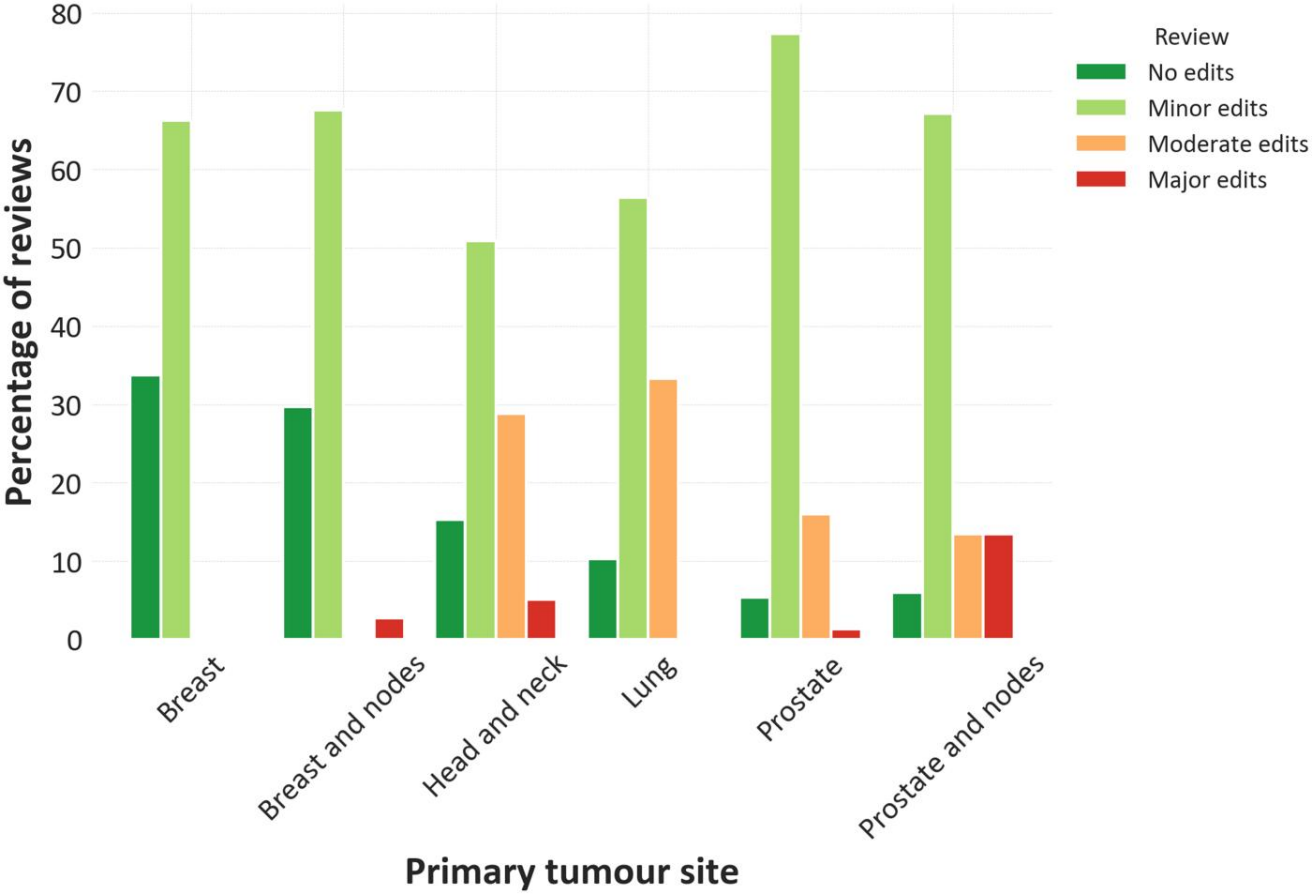
Frequency of acceptability scores by Reviewer 1 for AI-generated contours by tumour site.

Of the 396 AI-generated contour sets scored by Reviewer 1, 15.9% required no edits, 64.4% required minor edits, 16.2% required moderate edits, and 3.5% required major edits. Figure 5 shows that the acceptability scores broken down by tumour site. There was a significant association between tumour site and acceptability score (χ^2^=100.99, df = 15, *P* < .0001). Breast contours required least editing, with none requiring moderate or major adjustments, while prostate and head and neck contours required most editing. Of 184 AI-generated contours scored by Reviewer 2, 58% required no edits, 29% minor edits, 11% moderate edits, and 2% major edits.

## Discussion

This study aimed to assess the real-world effects of AI-assisted contouring on overall radiotherapy treatment planning workflows, staff allocation, and OAR contour acceptability. While AI-assisted pathways had generally shorter times from planning CT to contour completion, overall time savings were often lost at the point of contour review as review sessions were prebooked at regular intervals. Other activities taking place in the interval between contouring and reviewing were not formally recorded. In the manual pathway, clinical oncologists sometimes performed OAR contouring and target voluming simultaneously, while in the AI-assisted pathway, OAR review and target voluming might be combined when conducted by an oncologist, affecting the overall time savings depending on their availability. Local audit post-AI implementation could help to optimize pathways, for example to increase the proportion of cases meeting the waiting time standard, reduce overall pathway duration, create time for other activities, or address specific resource bottlenecks. In the manual pathway, oncologists were responsible for 86% of OAR contouring and all reviews. By eliminating the need for manual contouring and supporting reallocation of staff performing OAR contour review, AI not only streamlined the OAR contouring process but also enabled the redistribution of reviewing tasks across dosimetrists, therapeutic radiographers, medical physicists, and oncologists. This shift in workload distribution reduces the heavy reliance on oncologists, alleviating some of the pressures contributing to chronic burnout, out-of-hours working, and workforce attrition.^11^ Distributing elements of the contouring workflow to other professional groups offers flexibility to configure pathways and processes based on local requirements and staff availability, as well as the ability to advance practice and offer career development for some professions.

It is important to note that reduction of overall pathway duration is only one measure of efficiency. This study did not specifically report the number or proportion of cases that met the cancer waiting time standard. Interviews with staff at participating centres revealed that AI-assisted contouring could be deployed to help increase the proportion of cases processed within the standard timeframe, or that time savings extra to the standard could be invested in other activities such as service development and training. It should be noted that most departments had not formally reviewed and revised pathways after AI implementation.

The acceptability of AI-generated contours was also evaluated. Across the four primary tumour sites, 80% of OAR contour sets required no edits or only minor edits, indicating that the time saved through AI-assisted contouring was generally conserved during contour review. The need for edits varied by tumour site. Breast OAR contours required the fewest edits, while head and neck OARs required the most. Prostate and head and neck contours are more anatomically complex, often requiring several hours of manual contouring by an oncologist.^35–37^ As this evaluation focused on the impacts on efficiency and effectiveness at scale, the acceptability of contours was taken at this grouped anatomical level. However, a detailed exploration of the quality and reliability of contours, particularly in more complex anatomies, should ideally review constituent organs and sub-structures individually.

Furthermore, the performance of the AI depends on the quality of both the training datasets^38^^,^^39^ and the CT images.^40^ It is recognized that different AI tools perform better in some anatomical sites than others^21^^,^^31^^,^^42^ but this was not explored in this vendor-agnostic evaluation. Poorer acceptability in anatomies with smaller, elongated structures was corroborated in our data. A formal qualitative acceptability scoring was not performed for manual contours, as these represented standard clinical practice and were not independently re-evaluated using the same scale. Therefore, direct qualitative comparison between manual and AI-generated contour sets was not undertaken.

Previous evaluations of AI contouring have used granular “stopwatch” timing^41^^,^^42^ which is labour-intensive, prone to variation between users and departments, and was not practical for this evaluation. Instead, dates recorded in oncology information systems were used to provide a broader, system-level view of pathway impacts to generate recognizable real-world information. Although this approach masked finer details, such as out-of-hours activities or grouped tasks (e.g., batch uploads or multiple reviews), it reduced data collection burden and offered a more consistent comparison across departments. However, granular data would have been a valuable addition to explore the time taken for minor, moderate, and major contour edits. This study did not explore time to contour or edit per OAR because initial scoping work identified that departments used different local operating procedures with their own sets of OARs (e.g., relating to the type of radiotherapy or equipment available), and there was variation in the OARs reported by manufacturers of the commercial AI tools used.

A limitation of this study is that the Likert scale did not include an option for reviewers to determine that it would be preferable to reject AI-generated OAR contours and perform manual contouring from scratch rather than editing.

AI-assisted and manually contoured cases were not balanced in this real-world evaluation, with fewer manual cases concentrated in specific departments and sample sizes varying due to data availability. While statistical methods were used to adjust for these disparities,^43^ the inherent variability likely impacted the results. Additionally, two departments did not use AI during the study period. Although department was included as a covariate in models to adjust for institutional differences, structural workflow variation between fully manual and AI-adopting centres may have influenced comparisons.

An ideal prospective study would have featured standardized case selection, balanced groups and per OAR time and acceptability data collected for both AI and manually generated contours. However, collection of more granular data would have been both invasive and burdensome given the duration of the study and pressures facing departments. Such data, if collected in a rigorous and standardized manner within and between departments, would be a valuable addition to the knowledge base.

This multi-centre real-world evaluation was designed with a pragmatic approach intended to support participation by multiple radiotherapy departments across NHS England. It was designed to reflect routine practice to reduce bias and maximize the relevance of findings and generalizability to other such departments. As a result, heterogeneity in workflow, staffing, and case mix was both expected and intentional.

This research showed that differences in resource allocation and workflow mapping affected the benefits of AI-assisted contouring across the pathway. Departments should conduct workflow mapping before deploying AI, considering factors like tumour site distribution, staff availability for reviewing AI results, and potential bottlenecks. Clear governance, stakeholder involvement, and customized training are also needed for successful implementation.^44^ While AI saves time on specific tasks, ensuring that these savings are realised and utilized effectively requires ongoing auditing and workflow adjustments.

It should be noted that the data collection period for this study coincided with serious pressures on the radiotherapy workforce,^9^^,^^10^^,^^11^ a steady increase in cancer cases, and the pandemic and pandemic-related cancer service recovery. As current targets require that 96% of patients begin their first treatment within 31 days of a decision to treat, increased efficiency in a treatment planning workflow could manifest as shorter times to treatment for individual patients or as increasing proportions of patients being treated within this window. Accordingly, future studies could explore both the impact to time per patient and the volume of patients treated within a given timeframe, and the proportion treated within the 31-day target.

## Conclusion

AI-assisted OAR contouring offers potential efficiencies in radiotherapy treatment planning, particularly by reducing the time required for contouring and redistributing workload across non-oncologist staff. This has the potential to enable departments to focus staff at locally relevant bottlenecks to reduce overall pressure on the system. In this work, we found that AI significantly reduced time for contouring OARs, but the saving was not translated across the treatment planning workflow. Delays in contour review, for example related to staff availability or latencies in unoptimized pathways, limit the full realization of time savings across the workflow. Further work is required to understand this and to describe the other activities staff may have performed with time released by AI contouring tools that were not captured in our evaluation. While local post-implementation audit could help realize wider benefits of implementing AI auto-contouring, such activities require sustained time and resource investment and may not be feasible in settings where AI tools are deployed under short-term funding or contractual arrangements. AI-generated contours are generally acceptable across most tumour sites. Nonetheless, the greater variability in performance observed for complex anatomies such as head and neck cancers underscores the need for continued refinement of AI tools and processes to ensure consistent outcomes across all tumour sites for all patients. This study did not capture a granular measurement of the time taken to edit contours, nor a geometric quantification of how contours requiring minor, moderate, and major edits differed from ideal contours. Future work could explore geometric differences between unedited and edited contours and how the dosimetric consequences vary by OAR.

Further research on acceptability with clinicians and patients, as well as health economic modelling, is needed to assess the long-term impact and cost-effectiveness of AI-assisted contouring and extract health, time, morale, and effort savings for all stakeholders.
